# Exploring *Lachancea thermotolerans* biodiversity: insights into lactic acid production and carbon metabolism in response to oxygen availability

**DOI:** 10.1128/aem.01155-26

**Published:** 2026-08-05

**Authors:** Theo R. Jacobs, Mathabatha E. Setati, Carole Camarasa, Benoit Divol

**Affiliations:** 1 South African Grape and Wine Research Institute, Stellenbosch University 26697, Stellenbosch, South Africa; 2 UMR SPO, University of Montpellier, INRAE, Montpellier SupAgro 27037, Montpellier, France; Universita degli Studi di Napoli Federico II, Portici, Italy

**Keywords:** *Lachancea thermotolerans*, lactic acid production, oxygen availability, carbon metabolism, fermentation performance, wine, strain variability

## Abstract

Lactic acid production by *Lachancea thermotolerans* offers a promising biological strategy to restore wine acidity often compromised by climate change, yet this metabolic trait remains highly variable and poorly controlled. This study systematically evaluated the impact of controlled oxygenation regimes on fermentation performance and primary metabolism in three genetically distinct *L. thermotolerans* strains. Oxygen availability drastically altered metabolic fluxes. Under continuous aerobic conditions, carbon was redirected toward biomass formation (9- to 13-fold increase), with reduced ethanol and lactic acid yields, as well as increased succinic acid, acetoin, and 2,3-butanediol, whereas fully anaerobic conditions lowered fermentation performance but substantially increased lactic acid. These results confirmed that *L. thermotolerans* is a weakly Crabtree-positive yeast with mixed respiratory-fermentative metabolism. Carbon redistribution under aerobic conditions was not fully compensated by measured metabolites, resulting in substantially decreased carbon recovery (~70%) and suggesting undetermined respiratory CO_2_ production alongside diversion of carbon toward undetermined intracellular components. This imbalance was supported by a decline in cell density following peak growth, consistent with potential redox imbalance or oxidative stress. Lactic acid production occurred primarily under oxygen limitation, independent of ethanol formation, indicating a regulated, facultative adaptive response rather than a constitutive fermentation product. Strain-dependent differences further revealed distinct carbon allocation strategies. While oxygen strongly influenced metabolic behavior, pulse strategies showed limited, condition-dependent effects, highlighting the importance of timing and intensity for modulating lactic acid production. Overall, oxygen is a powerful lever to modulate metabolic fluxes in *L. thermotolerans*, but its application for predictable bio-acidification requires further optimization.

**IMPORTANCE:**

Wine acidity is increasingly difficult to maintain under climate change, creating a need for reliable biological solutions. Some strains of *Lachancea thermotolerans* can produce lactic acid during fermentation, but this metabolic trait is inconsistent and difficult to control. This study shows that oxygen availability is a key factor shaping how this yeast uses carbon, shifting it between growth and lactic acid production. Under the continuous aerobic condition, carbon flux was redirected toward biomass, whereas the fully anaerobic condition favored lactic acid production but reduced fermentation performance. Importantly, pulsed oxygen additions did not provide consistent control of lactic acid production, but they revealed trends that highlight the importance of timing and intensity. These findings show that lactic acid production is a flexible response to environmental conditions rather than a constitutive trait. This work provides a foundation for developing strategies to better control lactic acid production through timed oxygen addition during fermentation.

## INTRODUCTION

Acidity is critical for wine freshness, organoleptic balance, and microbial stability. Organic acids in wine are primarily derived from the grapes, although yeast metabolism during alcoholic fermentation can further modify the final acid profile. However, climate change presents a significant challenge, as rising global temperatures accelerate grape ripening and reduce natural acidity at harvest, particularly in warm-climate regions (1–3). To compensate, winemakers frequently use chemical acidification. Although effective, these practices increase production costs, may cause stability issues such as tartrate crystallization, and are increasingly rejected by consumers and regulatory bodies (3–6). This has created an urgent need for sustainable, biological solutions to maintain acidity under climate change conditions.

While *Saccharomyces cerevisiae* remains the dominant yeast species in winemaking, interest in non-*Saccharomyces* yeasts has increased due to their interesting metabolic traits. In this context, *Lachancea thermotolerans* has emerged as a leading candidate for biological acidification. It is the only yeast species known to produce substantial levels of lactic acid during alcoholic fermentation (7). Several studies have shown that certain strains can produce high concentrations of lactic acid, thereby naturally increasing wine acidity (7–10). In addition, this species has been associated with modest reductions in ethanol, volatile acidity, and malic acid, as well as improved aroma complexity and diverse sensory profiles (7, 8, 10–13). These traits make *L. thermotolerans* an attractive biological agent for climate-adaptive winemaking.

Despite these promising characteristics, a major limitation restricts the reliable application of *L. thermotolerans*: these traits, particularly lactic acid production, are highly variable between strains (7, 10, 13). Furthermore, even within the same strain, lactic acid levels can vary considerably under different environmental conditions, and this remains poorly understood (3, 7, 8). Reports show that some strains produce negligible amounts (<0.2 g/L), whereas others yield excessive concentrations (>20 g/L), in some cases resulting in sensorially unbalanced wines (7–10, 14, 15). As the underlying metabolic mechanisms are not yet clearly defined, production is currently unpredictable and difficult to control, limiting the consistent industrial use of this species. To date, research has largely focused on identifying conditions that enhance lactic acid production, rather than understanding how to regulate this trait to achieve consistent and targeted acidification, which is the real challenge.

Several oenologically relevant environmental factors have been shown to influence lactic acid production in *L. thermotolerans*. Lower fermentation temperatures prolong yeast persistence and indirectly increase lactic acid levels (16). Nitrogen limitation has been associated with increased lactate production (17), and inoculation strategies in co-culture with *S. cerevisiae* significantly alter both yeast persistence and lactic acid levels (18). A recent comprehensive study using synthetic must and transcriptomic analysis demonstrated that high sugar concentrations, elevated pH, and low organic nitrogen upregulate glycolytic and lactate dehydrogenase (*LDH*) genes, increasing metabolic flux toward lactic acid (3). However, most studies have relied on co-culture systems or evaluated a single strain, making it difficult to disentangle intrinsic metabolic responses from effects driven by interspecies interactions or strain-specific behavior.

Oxygen availability represents a particularly intriguing yet insufficiently resolved factor. It influences yeast physiology, redox balance, and carbon flux distribution. In co-culture fermentations, oxygen supply enhances *L. thermotolerans* growth and persistence (19, 20). Transcriptomic analysis further revealed strong upregulation of *LDH* genes under anaerobic conditions (21), suggesting a potential shift of carbon flux toward lactate. Another study (22) showed substantially higher lactic acid production under anaerobic compared which semi-aerobic conditions, accompanied by compensatory increases in ethanol and biomass when oxygen was available. However, previous studies presented at least one of the following shortcomings: (i) they did not directly quantify lactic acid, (ii) oxygen exposure was not quantitatively defined, controlled, or monitored (e.g., no measurement of dissolved oxygen levels), (iii) they were performed in co-culture systems, or (iv) they examined only a single strain, which was a non-lactic acid producer. Therefore, current understanding of how oxygen shapes carbon metabolism and lactic acid production across genetically distinct *L. thermotolerans* strains remains limited.

The aim of this study was to systematically assess the impact of controlled oxygenation regimes on fermentation performance and primary metabolite dynamics, with a specific focus on lactic acid production, across three genetically distinct *L. thermotolerans* strains exhibiting variable production capacities in monoculture fermentations to better understand and potentially control this promising but highly variable trait for more predictable, sustainable wine production.

## MATERIALS AND METHODS

### Yeast strains and growth conditions

Three *L. thermotolerans* strains, previously confirmed as genetically distinct by microsatellite typing (13), were selected: Y548 (isolated from Cabernet Sauvignon grape must, Stellenbosch, 2020), Y519 (isolated from Cabernet Sauvignon grape must, Stellenbosch, 2018), and Y1240 (isolated from Muscat of Alexandria grape must, Rawsonville, 2009). These strains, which belong to the microbial culture collection of the South African Grape and Wine Research Institute, Stellenbosch University, were chosen to cover the full range of lactic acid production. Indeed, in our previous work, Y1240 was identified as a non-producer (also reported in references 21, 23), Y519 as an intermediate producer, and Y548 as a high producer (13). All strains were propagated from cryopreserved cultures stored at −80°C in 25% (vol/vol) glycerol by streaking onto sterile Wallerstein Laboratory Nutrient agar (Biolab-Merck, Modderfontein, South Africa), prepared according to the manufacturer’s instructions, and incubated at 30°C for 3 days to obtain single colonies.

### Preculture conditions

For pre-cultures, a single colony of each strain was inoculated into a test tube containing 5 mL of sterile yeast extract-peptone-dextrose (YPD) broth (1% yeast extract, 2% peptone, 2% glucose) and incubated at 30°C on a spinning wheel (40 rpm) for 14 h to reach the mid-exponential growth phase. Thereafter, each preculture was inoculated into 10 mL of fresh YPD broth and incubated for an additional 14 h at 30°C on a spinning wheel (40 rpm) to reach the mid-exponential growth phase again. Finally, yeast cultures were harvested by centrifugation at 615 × *g* for 5 min, rinsed with 0.9% NaCl, and inoculated into the synthetic grape juice (SGJ) medium at an OD_600_ (optical density at 600 nm) of 0.2 (i.e., approximately 1 × 10⁶ CFU/mL).

### Fermentation medium

SGJ, simulating standard grape must with a sugar concentration of 230 g/L, was prepared according to a previously published protocol (24). A yeast assimilable nitrogen concentration of 300 mg/L (25) was used as the total nitrogen. The full composition of the SGJ is provided in Table S1. The pH of the base SGJ medium, excluding the nitrogen, vitamin, and trace element stock solutions, was adjusted to 3.3 using 10 M NaOH prior to sterilization at 121°C for 15 min. The nitrogen, vitamin, and trace element stock solutions were filter-sterilized using a 0.22 μm syringe filter (Merck Millipore, Darmstadt, Germany) and added aseptically to the sterile SGJ.

### Fermentation conditions

Different oxygenation regimes were applied to evaluate the effect of oxygen availability on fermentation performance and metabolite production. All fermentations were performed in triplicate.

A control fermentation was designed to simulate typical industrial winemaking conditions, where grape must is exposed to oxygen during crushing and pressing and is therefore initially saturated with oxygen prior to the start of fermentation. The control fermentation was conducted in 1.2 L cylindrical fermenters equipped with sampling ports, fermentation airlocks equipped with bubblers to maintain anaerobic conditions, and a probe port for temperature monitoring. Each fermenter was filled with 1 L of SGJ. Prior to inoculation, the medium was sparged with sterile air through a 0.22 μm syringe filter using a sterile surgical needle inserted via the sampling port until oxygen saturation was reached (approximately 30 min). Dissolved oxygen levels were measured using a Fibox standalone fiber-optic oxygen meter (PreSens, Regensburg, Germany) with SP-PSt3-NAU oxygen sensor spots placed in the medium. Fermentations were conducted at 20°C. Fermenters were placed on automated balances connected to an online fermentation monitoring system called the “Alcoholic Fermentation Information System” (ALFIS, INRAE, Montpellier, France), which recorded weight loss every 20 min throughout fermentation. CO₂ production (g/L) and fermentation rates (g CO_2_/L/h) were calculated automatically from weight loss data, with corrections applied for evaporation and sampling losses. Temperature was also continuously monitored via a probe inserted into the fermenter. Fermentations were performed under continuous agitation (120 rpm) using magnetic stirrer bars until completion of fermentation. Agitation was applied to fermentations receiving oxygen treatments to ensure even distribution of dissolved oxygen throughout the fermentation medium, rather than to facilitate yeast suspension or mixing.

Fully anaerobic fermentations were conducted in 100 mL penicillin vials equipped with a single sampling port sealed with a rubber septum and cap. Each fermenter was filled with 90 mL of SGJ to minimize headspace and maintain anaerobic conditions. The use of 100 mL penicillin vials was chosen to achieve strict anaerobiosis, as such conditions are more easily maintained in small, sealed vessels than in 1 L fermenters. Preliminary experiments verified that yeast growth and fermentation performance are comparable between penicillin vials and 1 L fermenters under similar oxygenation conditions. Prior to inoculation, the medium was sparged with sterile argon, in the same manner as described above, until dissolved oxygen levels were below the detection limit (<0.001 mg/L). Preliminary trials indicated that reaching this level required approximately 3 h. Fermentations were conducted at 20°C without agitation. Fermenters were initially sealed to maintain anaerobic conditions; however, after approximately 48 h, visible yeast growth necessitated allowing CO₂ release to prevent pressure build-up. Uninoculated fermenters containing SGJ were included as controls to account for evaporation. Fermentations were monitored by manual weighing every 24 h, and CO₂ release was calculated from weight loss, with corrections applied for evaporation and sampling.

Fully aerobic fermentations were conducted in 1 L Erlenmeyer flasks sealed with sterile cotton to prevent contamination while allowing continuous oxygen entry. Each flask was filled with 250 mL of SGJ to provide a large headspace and promote aerobic conditions. Prior to inoculation, the medium was sparged with sterile air as described above until oxygen saturation was reached. Fermentations were conducted at 20°C with continuous high-speed stirring using magnetic stirrer bars to ensure thorough oxygen distribution throughout fermentation. Continuous aeration using a needle connected to the air system and the automated weighing system (ALFIS) was not feasible for this treatment due to technical constraints. Alternative measures were implemented to maintain oxygen saturation throughout fermentation. Flasks were sealed with cotton for passive oxygen diffusion, the large headspace was maintained, and flasks were opened daily under sterile conditions in a laminar flow hood to refresh the air. Dissolved oxygen levels were monitored continuously to confirm that the medium remained saturated from start to finish. Uninoculated fermenters containing SGJ were included as controls to account for evaporation. Fermentations were monitored by manual weighing every 24 h, and CO₂ release was calculated from weight loss, with corrections applied for evaporation and sampling.

Pulse oxygenation treatments were conducted under the same conditions as the control fermentations described above. The SGJ was saturated with oxygen prior to inoculation, after which additional oxygen pulses were applied at specific stages of fermentation to evaluate the effect of oxygen exposure at different physiological phases. Oxygen pulses were applied by sparging sterile air into the fermenters until oxygen saturation was reached. The additional pulses were applied at one of the following fermentation stages, determined from the fermentation kinetics and optical density (OD₆₀₀) of the control fermentation: (i) 20 h after inoculation, corresponding to the end of the lag phase; (ii) at 25% of maximum optical density (OD₆₀₀), corresponding to the point of maximum fermentation rate; or (iii) after 40 g/L CO₂ loss, corresponding to the onset of the stationary phase. Apart from the timing of the additional oxygen pulses, all fermentation conditions and monitoring procedures were identical to those described for the control fermentations.

All three strains were used, except for the pulse oxygenation treatments, which were conducted only with the two strains representing the extremes of lactic acid production, namely the non-lactic acid-producing strain Y1240 and the high lactic acid-producing strain Y548.

### Sampling and monitoring of fermentation

Fermentation kinetics were monitored by measuring weight loss over time, which served as a proxy for CO₂ release. As described above, control and pulse oxygenation fermentations were monitored using analytical balances connected to ALFIS, which recorded weight loss every 20 min from the start to the end of fermentation. Fully aerobic and fully anaerobic fermentations were weighed manually every 24 h to record weight loss. Cumulative CO₂ production was calculated from weight loss data, with corrections applied for evaporation and sampling losses. Since *L. thermotolerans* strains in monoculture typically do not complete alcoholic fermentation, fermentations were terminated when fermentation rates dropped below 0.01 g CO_2_/L/h. Yeast growth was monitored daily by measuring OD₆₀₀ using a VIS spectrophotometer (E-SP1000VP, Eins-Sci, Adelaide, Australia) from the start of fermentation until OD values stabilized or began to decline. At the end of fermentation, yeast growth was also determined by measuring dry biomass, whereby cells were harvested by centrifugation, washed, dried to constant weight, and weighed. For chemical analysis, 5 mL samples were taken every 24 h from the beginning to the end of fermentation through the sampling port using a sterile needle and syringe. Samples were centrifuged at approximately 4,000 × *g* for 5 min using a UNIVERSAL 320 benchtop centrifuge (Hettich, North America) equipped with a swing-out rotor. The resulting supernatant was collected and stored at −20°C until further analysis.

### Chemical analyses

#### Quantification of major sugars, organic acids, glycerol, and ethanol

The concentrations of major sugars (glucose, fructose), organic acids (lactic, acetic, and succinic acids), glycerol, and ethanol were quantified using high-performance liquid chromatography (HPLC 1260 Infinity, Agilent Technologies, Santa Clara, CA, USA) equipped with a Phenomenex Rezex ROA column (Phenomenex, Le Pecq, France) maintained at 60°C. The mobile phase consisted of 0.005 N H₂SO₄ at a flow rate of 0.6 mL/min. Prior to HPLC analysis, samples were diluted fivefold to ensure accurate quantification due to the high sugar concentrations present at the beginning of fermentation and the residual sugars present at the end of fermentation. Organic acids, with the exception of succinate, were detected using a UV detector at 210 nm, while the remaining compounds, including succinate, were detected using a refractive index detector (Agilent Technologies). Quantification was performed using external calibration curves generated from analytical standards by plotting peak area against concentration. Data acquisition and chromatographic analysis were performed using OpenLab CDS software (Agilent Technologies).

#### Quantification of acetoin and 2,3-butanediol

Acetoin and 2,3-butanediol were quantified only in the contrasting oxygenation treatments at the end of fermentation by gas chromatography coupled with flame ionization detection. Samples were first subjected to a double liquid-liquid extraction using chloroform, with 1 mL of hexanol (1:1,000 vol/vol) added as an internal standard. The resulting organic phase was dried before injection into the GC column. Analyses were carried out using an HP 5890 gas chromatograph (Agilent Technologies) fitted with an SGE BP20 polar column (30 m × 0.53 mm × 1.0 µm) and coupled to a flame ionization detector.

### Data processing and statistical analyses

Data analysis was performed separately for the contrasting oxygenation treatments (fully anaerobic, oxygen saturation before inoculation [control], and fully aerobic) and the pulse oxygenation treatments (oxygen saturation before inoculation [control], additional oxygen pulse at 20 h of fermentation, at 25% maximum OD, or at 40 g/L CO₂ loss) to simplify interpretation and comparison of results.

Descriptive statistics (means and standard deviations) were calculated to assess variability among biological replicates. Fermentation kinetics were presented as cumulative CO₂ loss (g/L) and yeast growth as optical density (OD₆₀₀) over time (h). Line graphs were generated using Microsoft Excel (version 16.93.1, Washington, USA).

Primary metabolite production during fermentation was visualized using heatmap analysis to simplify interpretation of time-course data collected at 24 h intervals throughout fermentation. To further standardize comparisons between fermentations with different durations, metabolite concentrations were evaluated at specific fermentation progress points corresponding to 25% increments of total CO₂ loss (0%–100%). For heatmap generation, metabolite concentrations were standardized using Z-scores calculated from measured concentrations (g/L) to allow comparison between metabolites with different concentration ranges. Yield-based normalization was not applied because sugar quantification at early fermentation stages was inaccurate due to the very high sugar concentrations in synthetic grape juice, which limited reliable HPLC quantification. Pearson correlation analysis was performed to assess relationships between metabolites. Heatmaps were generated, and correlation analyses were performed using the PAST software (version 4.15; 26).

Endpoint metabolite data were expressed as yields (mol/mol sugar consumed). These data were visualized as bar graphs generated using Microsoft Excel and as a simplified metabolic pathway representation of alcoholic fermentation. An additional figure showing metabolite production relative to biomass was generated to account for differences in biomass production between treatments. Statistical analyses were performed using XLSTAT software (version 2025.1, Paris, France). One-way analysis of variance was used to determine significant differences, first within each strain across oxygenation treatments, and then between strains within each oxygenation treatment. Normality of data distribution was verified prior to analysis. Tukey’s HSD test was used for pairwise comparisons at a 95% confidence level (*P* < 0.05). Principal component analysis (PCA) was performed using XLSTAT to evaluate the relationships between strains, oxygenation treatments, and primary metabolite production. Results were presented as biplots. All graphs and figures were generated using the following software: Microsoft Excel, XLSTAT, and PAST. Carbon balance was also calculated on a carbon-mole (C-mol) basis. C-mol values were obtained by multiplying the molar concentration of each metabolite by the number of carbon atoms in the respective compound (e.g., ethanol × 2, glycerol × 3, lactic acid × 3, acetic acid × 2, succinic acid × 4, acetoin × 4, 2,3-butanediol × 4, and residual glucose/fructose × 6). Total product C-mol was calculated as the sum of all measured metabolites, including CO₂ estimated from ethanol assuming a 1:1 molar ratio. Carbon recovery (%) was calculated as (total product C-mol / consumed sugar C-mol) × 100, where consumed sugar C-mol was calculated as [(initial glucose + initial fructose) − (residual glucose + residual fructose)] × 6.

## RESULTS

### Fermentation performance under different oxygenation strategies

#### Contrasting oxygenation treatments

**Fig 1:**
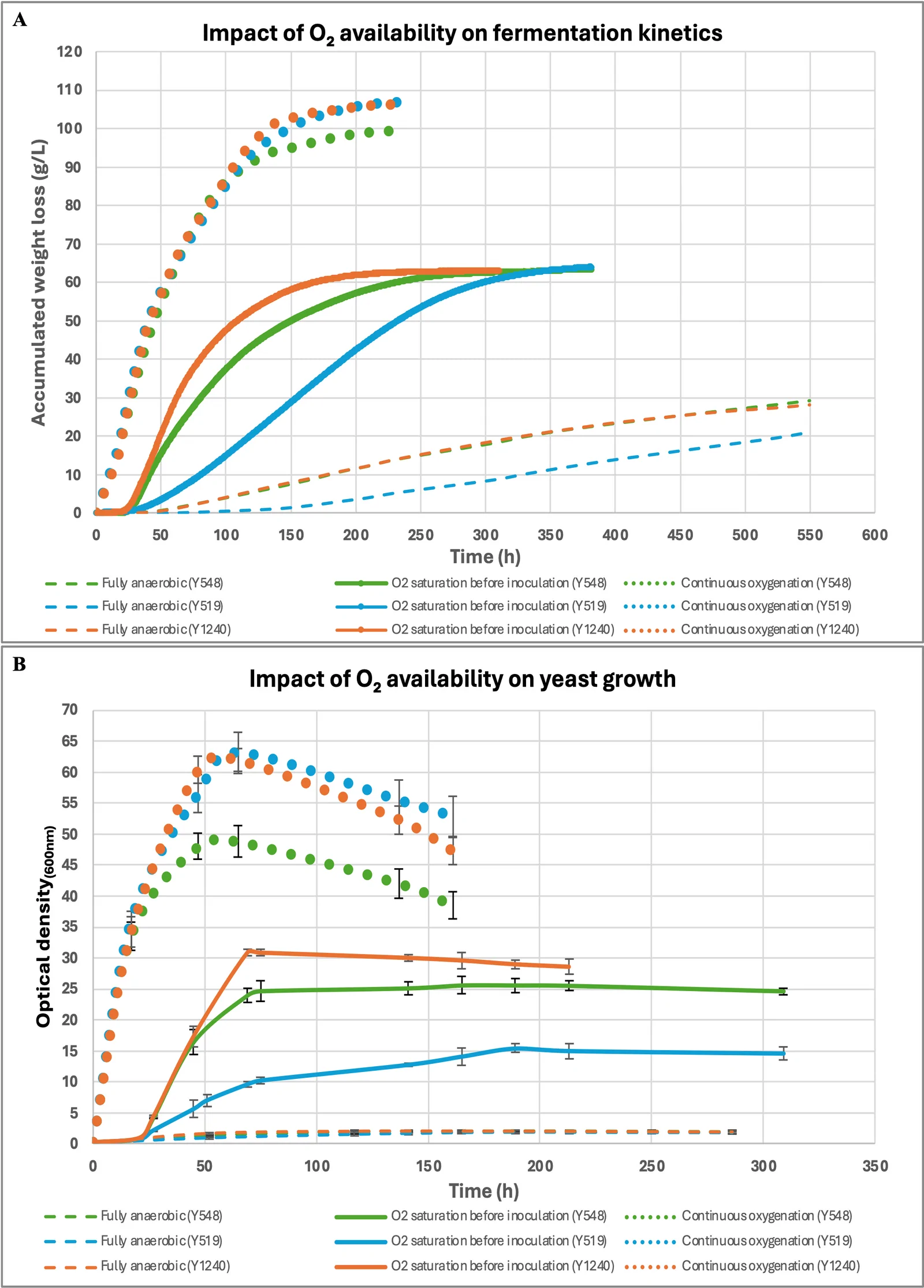
Fermentation performance of three genetically distinct *Lachancea thermotolerans* strains under different oxygenation regimes in synthetic grape juice (230 g/L sugar). (**A**) Fermentation kinetics expressed as accumulated weight loss (g/L) due to CO₂ release, representing mean values of triplicate fermentations. (**B**) Yeast growth monitored by optical density at 600 nm (OD₆₀₀). Oxygen treatments included fully anaerobic conditions, oxygen saturation before inoculation (control), and continuous oxygenation throughout fermentation. Different colors represent the three strains (Y548: green; Y519: blue; and Y1240: orange), while line types represent the oxygen treatments (dashed line: fully anaerobic; solid line: oxygen saturation before inoculation [control]; and dotted line: continuous oxygenation). Data points represent mean values of triplicate fermentations, and error bars indicate standard deviation. Standard deviation is not shown for fermentation kinetics due to the high-frequency automated weighing (every 20 min) for the oxygen saturation before inoculation (control), which would overcrowd the figure. The relative standard deviation remained below 5% across triplicate fermentations, indicating good reproducibility. Raw fermentation kinetics data underlying the CO₂ production curves are provided in Table S8 (Fermentation kinetics raw data).

The fermentation performance of three genetically distinct *L. thermotolerans* strains, Y548 (high lactic acid producer), Y519 (intermediate lactic acid producer), and Y1240 (non-lactic acid producer), was evaluated under three distinct oxygenation strategies, including fully anaerobic conditions, oxygen saturation before inoculation (control), and continuous oxygenation throughout fermentation. Fermentation kinetics were monitored by CO₂ release, while yeast growth was assessed by OD₆₀₀ (Fig. 1).

Clear differences in fermentation performance were observed between oxygenation treatments, with additional strain-specific differences within each treatment (Fig. 1A). Under fully anaerobic conditions, fermentation resulted in only approximately 20%–30% of the maximum CO₂ loss after 550 h for all strains. Under the oxygen saturation before inoculation (control) treatment, representing standard industry-relevant conditions, fermentation progressed further but remained incomplete, reaching approximately 60% CO₂ loss. Under both conditions, strains Y548 and Y1240 displayed similar fermentation kinetics, whereas Y519 showed a slower fermentation rate and a delayed onset of fermentation activity. In contrast, continuous oxygenation substantially improved fermentation performance. Under this treatment, the fermentation pattern changed: strains Y519 and Y1240 showed the highest cumulative weight loss, while Y548 showed lower weight loss. Residual sugar concentrations confirmed that Y519 and Y1240 completed fermentation (<1 g/L), whereas Y548 retained approximately 9 g/L residual sugar (Table S2).

Trends for yeast growth mirrored those observed for the fermentation kinetics (Fig. 1B). Under fully anaerobic conditions, growth was limited for all strains, and the OD_600_ remained relatively constant throughout fermentation, only reaching a maximum of approximately 2.5 and showing little variation between strains. Under the oxygen saturation before inoculation (control) treatment, higher cell densities were observed, and strain-specific differences became apparent. Strain Y1240 reached the highest maximum OD₆₀₀ (~30), followed by Y548 (~25), while Y519 reached the lowest maximum OD₆₀₀ (~15). Under this oxygen saturation before inoculation (control) treatment, OD₆₀₀ reached a maximum at approximately 72 h and then gradually declined toward the end of fermentation. Continuous oxygenation resulted in substantially higher cell densities. Under these conditions, Y519 and Y1240 reached comparable maximum OD₆₀₀ values (~65 and ~62, respectively), while Y548 reached a lower maximum OD₆₀₀ (~50), consistent with the trends observed for weight loss due to CO_2_ release. Like the oxygen saturation before inoculation (control) treatment, maximum OD₆₀₀ values were reached at approximately 72 h. However, in contrast to the gradual decline observed under the oxygen saturation before inoculation (control) treatment, OD₆₀₀ decreased rapidly thereafter, indicating a rapid loss of cell density while fermentation was still ongoing. Despite this early and rapid decline in cell density, sugar consumption was complete under continuous oxygenation (Table S2).

#### Timed oxygen pulse treatments

**Fig 2:**
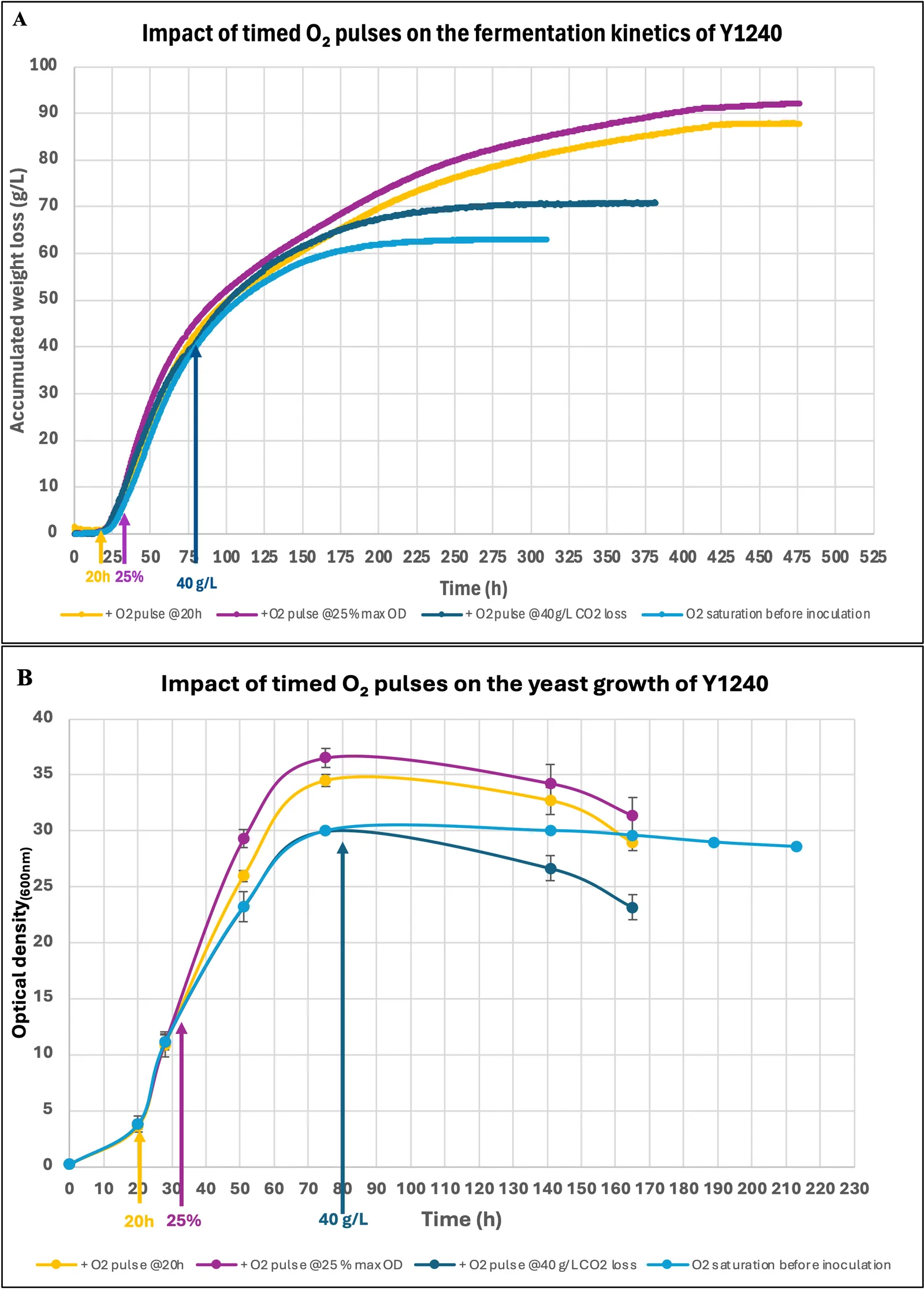
Fermentation performance of *Lachancea thermotolerans* strain Y1240 under timed oxygen pulse treatments in synthetic grape juice (230 g/L sugar). (**A**) Fermentation kinetics expressed as accumulated weight loss (g/L) due to CO₂ release, representing mean values of triplicate fermentations. (**B**) Yeast growth monitored by optical density at 600 nm (OD₆₀₀). Oxygen treatments included oxygen saturation before inoculation (control, blue), oxygen saturation before inoculation followed by an oxygen pulse at 20 h of fermentation (yellow), at 25% of maximum OD (purple), or at 40 g/L CO₂ loss (dark blue). Data points represent mean values of triplicate fermentations, and error bars indicate standard deviation. Standard deviation is not shown for fermentation kinetics due to the high-frequency automated weighing (every 20 min), which would overcrowd the figure. Raw fermentation kinetics data underlying the CO₂ production curves are provided in Table S8 (Fermentation kinetics raw data).

The impact of timed oxygen pulses on fermentation performance was evaluated for the two strains representing the extremes of lactic acid production, Y1240 (Fig. 2) and Y548 (Fig. S1). In all treatments, the synthetic grape juice was saturated with oxygen prior to inoculation, followed by additional oxygen pulses applied at different fermentation stages: 20 h after inoculation (around 5% of maximum OD), at 25% of maximum OD (maximum fermentation rate), or at 40 g/L CO₂ loss (maximum OD).

Timed oxygen pulses affected fermentation kinetics relative to the oxygen saturation before inoculation (control) treatment, which received oxygen only before inoculation. An oxygen pulse at 40 g/L CO₂ loss only slightly increased maximum CO₂ loss compared to the oxygen saturation before inoculation (control), by about 8 g/L for both strains (Fig. 2A and Fig. S1A). In contrast, a pulse at 20 h led to a larger increase: approximately 25 g/L for Y1240 and 15 g/L for Y548 (Fig. 2A and Fig. S1A). The largest increase occurred with a pulse at 25% of maximum OD, raising maximum CO₂ loss by about 30 g/L for Y1240 and 18 g/L for Y548 relative to the oxygen saturation before inoculation (control).

Similar trends were observed for yeast growth (Fig. 2B). An oxygen pulse at 40 g/L CO₂ loss had no effect on maximum OD. However, pulsing at 20 h increased OD by ~5 units for both strains (Y1240: Fig. 2B; Y548: Fig. S1B). The largest increases occurred at 25% of maximum OD, raising OD by ~7 units for Y1240 (Fig. 2B) and 10 units for Y548 (Fig. S1B) relative to the oxygen saturation before inoculation (control). While the oxygen saturation before inoculation (control) OD₆₀₀ peaked at around 72 h and then gradually declined, oxygen-pulsed fermentations peaked at the same time but showed a more rapid decline thereafter.

### Primary metabolite dynamics under different oxygenation strategies

**Fig 3:**
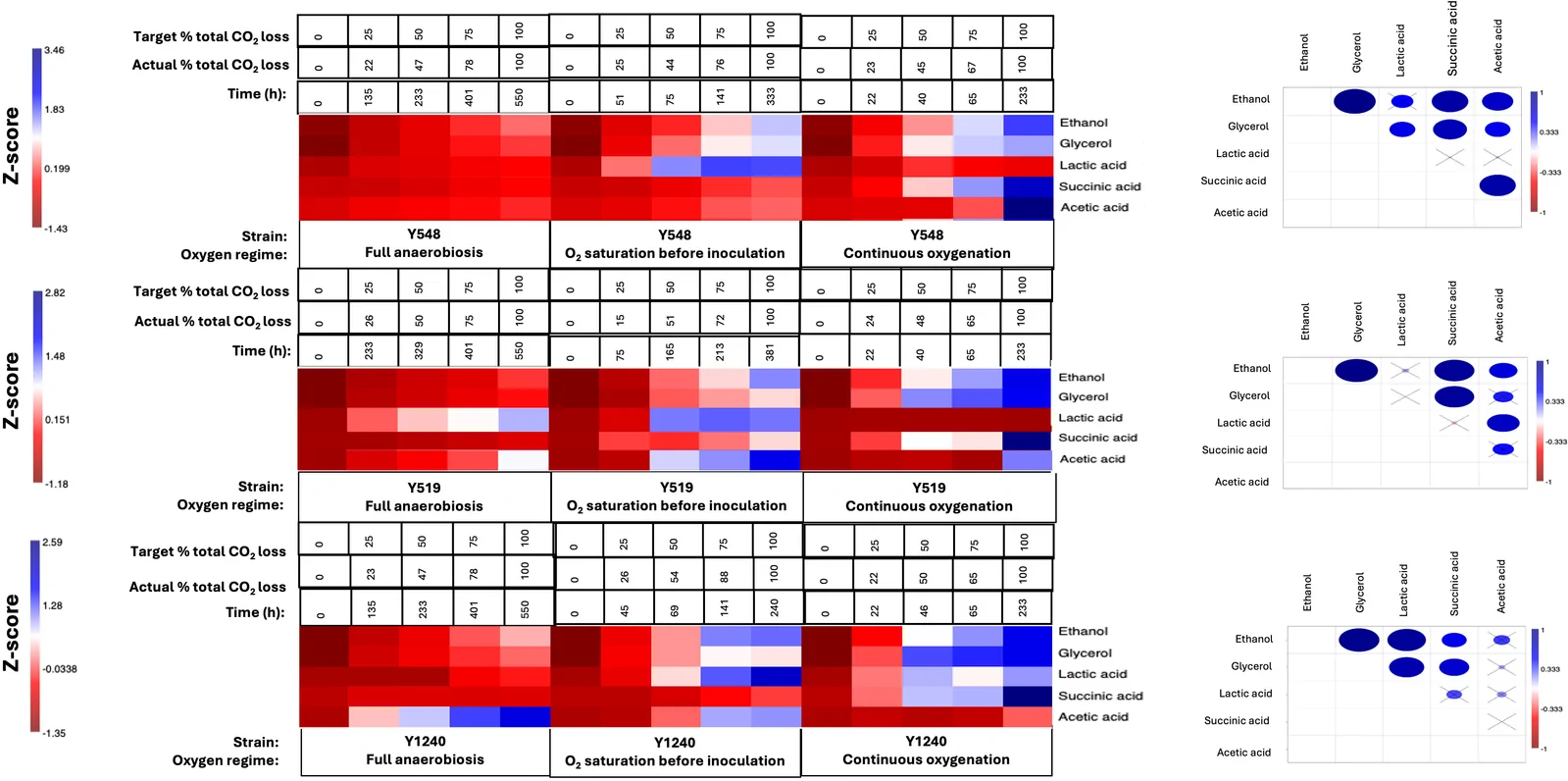
Heatmap representation of primary metabolite dynamics during alcoholic fermentation of *Lachancea thermotolerans* under contrasting oxygenation conditions in synthetic grape juice. Heatmaps are presented strain-by-strain to illustrate the effect of oxygen treatment within each strain. Metabolite concentrations (g/L) were standardized using Z-scores to facilitate comparison between metabolites. Z-scores were calculated separately for each metabolite within each strain using the mean and standard deviation across all oxygenation conditions and time points, allowing comparison of relative changes between oxygenation treatments within a given strain. Color intensity reflects relative metabolite abundance, with red indicating lower relative concentrations and blue indicating higher relative concentrations. Data are presented at selected fermentation stages corresponding to approximately 25% increments of total CO₂ loss, with the associated sampling time and actual CO₂ loss indicated. For each strain and oxygenation treatment, 100% total CO₂ loss was defined as the maximum cumulative CO₂ loss reached during that fermentation. Target sampling stages (25, 50, 75, and 100%) were defined as proportions of this maximum, and samples were assigned to the closest actual CO₂ loss achieved. Pearson correlation analysis was performed using metabolite concentrations across all oxygenation conditions and time points within each strain. Correlation coefficients are shown adjacent to the heatmaps, with bubble size proportional to the Pearson correlation coefficient, and non-significant correlations (*P* > 0.05) are crossed out. Raw metabolite concentration data underlying this figure are provided in Table S9 (Primary metabolite raw data).

Primary metabolite dynamics during alcoholic fermentation were visualized using heatmap analysis to compare metabolite production patterns across oxygenation treatments and strains (Fig. 3 and Fig. S2 and S3). In addition, Pearson correlation analysis was performed to evaluate relationships between metabolites and identify associations in their production patterns across oxygenation treatments and strains.

#### Contrasting oxygenation treatments

Primary metabolite dynamics under contrasting oxygenation were evaluated for three strains (Fig. 3). The main heatmap is organized strain-by-strain to compare oxygenation conditions, whereas a treatment-by-treatment alternative is provided in Fig. S2 to compare strains. Both visualizations were based on Z-score standardization of metabolite concentrations (g/L).

Yield standardization could not be performed due to inaccurate sugar measurements at the beginning of fermentation, where sugar concentrations exceeded the reliable quantification range of the HPLC. Therefore, relative color intensity reflects trends within each data set rather than absolute metabolite concentrations. This is particularly evident for Y1240, where relative changes appear pronounced in the heatmap despite only trace lactic acid concentrations being detected.

Lactic acid production dynamics differed substantially between oxygenation treatments and strains (Fig. 3; Fig. S2 and Table S3). The highest lactic acid concentrations were observed under the oxygen saturation before inoculation (control) condition, where strains Y548 and Y519 reached concentrations of 11.2 g/L lactic acid and 15.5 g/L lactic acid, respectively. Lactic acid production under the oxygen saturation before inoculation (control) condition became clearly evident from the mid-stages of fermentation (approximately 50% of total CO₂ loss), and increased progressively until the end of fermentation. Nevertheless, fermentation progress was evaluated at discrete CO₂ loss intervals; therefore, the exact onset of lactic acid production may have occurred between the fermentation stages where 25% and 50% of the total CO_2_ had been released. The non-lactic acid-producing strain Y1240 showed only trace concentrations (0.01 g/L–0.05 g/L lactic acid) across all treatments, despite appearing relatively abundant in the heatmap as a result of Z-score standardization. Under fully anaerobic conditions, lactic acid production was reduced for Y548 compared to the oxygen saturation before inoculation (control) treatment, whereas Y519 still produced relatively high lactic acid concentrations (13.8 g/L), despite much lower overall sugar consumption under anaerobic conditions. Interestingly, lactic acid production for Y519 under anaerobic conditions began earlier in fermentation (approximately 25% of total CO₂ loss) compared to the oxygen saturation before inoculation (control) treatment and also increased until the end. Under continuous oxygenation, lactic acid production was largely suppressed. In contrast, succinic acid production was highest under continuous oxygenation across all strains, showing an inverse metabolic response compared to lactic acid.

**Fig 4:**
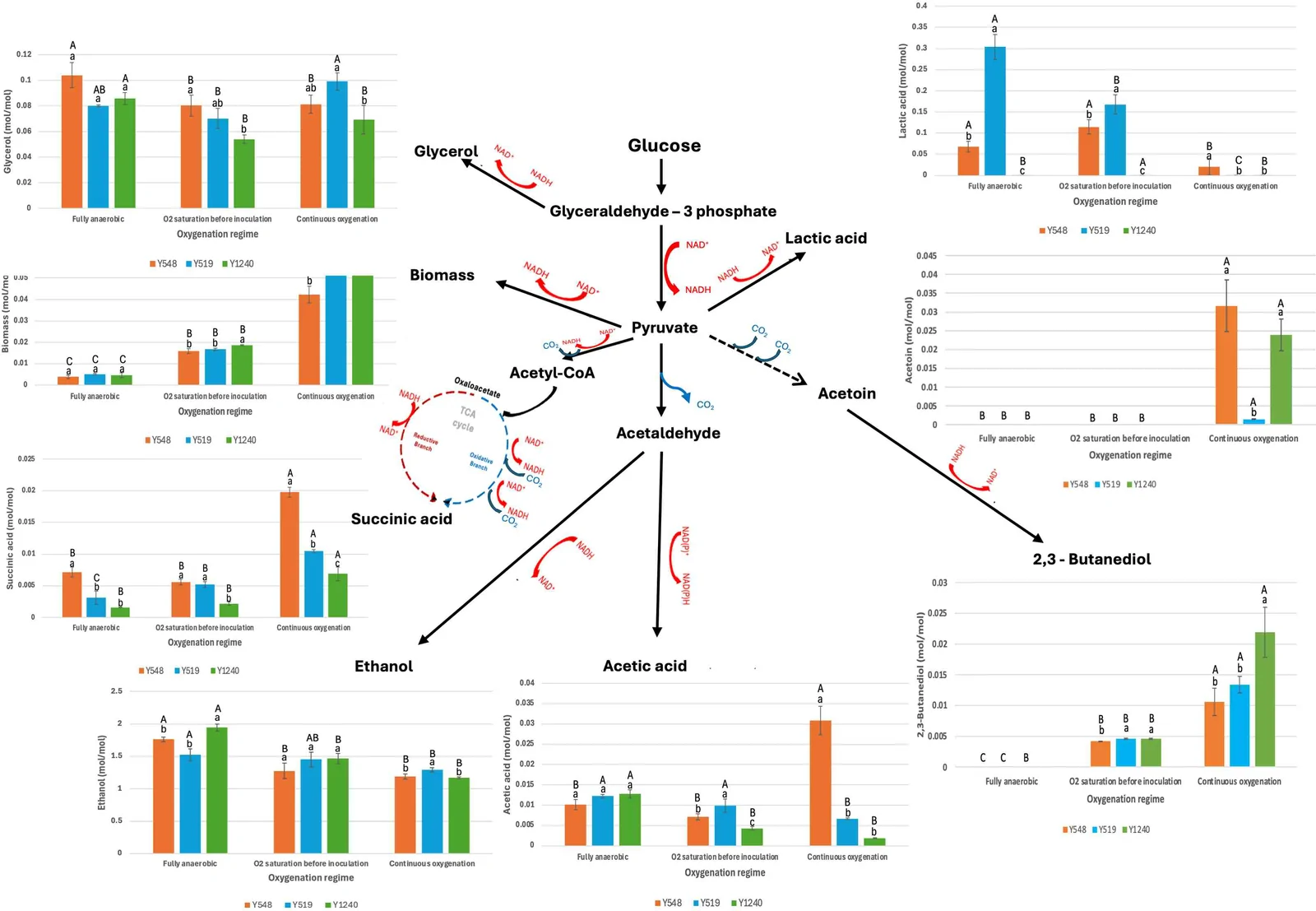
Simplified representation of central carbon metabolism in *Lachancea thermotolerans* during alcoholic fermentation under contrasting oxygenation conditions in synthetic grape juice. Metabolite production and biomass are expressed as yields (mol product per mol sugar consumed) at the end of alcoholic fermentation and displayed as bar graphs integrated within the metabolic pathway. Strains are grouped within each oxygen treatment. Bars represent mean values of triplicate fermentations, with error bars indicating standard deviations. Different letters above bars indicate statistically significant differences (*P* < 0.0001); uppercase letters denote differences within a strain across oxygen treatments, and lowercase letters denote differences between strains within the same oxygen treatment.

Comparison between strains within each treatment (Fig. S2 and Fig. 4) confirmed that lactic acid production was strain-dependent. Under both oxygen saturation before inoculation (control) and fully anaerobic conditions, Y519 produced the highest relative lactic acid concentrations, followed by Y548, while Y1240 showed negligible production (Fig. S2 and Fig. 4). Interestingly, Y519 exceeded Y548 in these treatments despite Y548 being previously identified as a higher lactic acid producer (13). Under continuous oxygenation, Y548 was the only strain that produced measurable lactic acid, and interestingly, production peaked during the mid-stages of fermentation (approximately 50% of total CO₂ loss) and then gradually declined toward the end of fermentation (Fig. 3). In addition to these differences, acetic acid profiles were strongly strain- and condition-dependent, with Y548 showing pronounced production only at the end of the continuous aerobic fermentation, Y519 exhibiting elevated levels under the oxygen saturation before inoculation (control) condition, and Y1240 displaying high relative production under the fully anaerobic condition (Fig. 3).

Correlation analysis revealed relationships between lactic acid and other metabolites that differed between strains and treatments. For Y548, lactic acid was positively correlated with glycerol across treatments, whereas for Y519, lactic acid showed a positive correlation with acetic acid. When analyzed by treatment, lactic acid was positively correlated with glycerol, succinic acid, and acetic acid under the oxygen saturation before inoculation (control) condition, while no clear correlations were observed under anaerobic conditions. Under continuous oxygenation, lactic acid showed a positive correlation with succinic acid. Interestingly, no correlation was observed between lactic acid and ethanol under any condition.

#### Timed oxygen pulse treatments

Primary metabolite dynamics under timed oxygen pulse treatments were evaluated for strains Y548 and Y1240 (Fig. S3).

Unlike the contrasting oxygenation treatments, pulsed oxygenation had minimal impact on primary metabolite dynamics compared to the oxygen saturation before inoculation (control) treatment. Lactic acid production by Y548 was not significantly affected by the oxygen pulses, with both the final concentrations (Fig. S3 and Fig. 4) and timing of production remaining similar to the oxygen saturation before inoculation (control). Lactic acid production was generally first detected between approximately 25% and 50% of total CO₂ loss and increased until the end of fermentation in all treatments, indicating that pulse timing had little influence on lactic acid production. As expected, Y1240 did not produce measurable lactic acid under pulse oxygenation treatments.

Oxygen pulses also had no measurable effect on ethanol and glycerol production. Acetic acid concentrations were slightly lower in the 20 h and 25% maximum OD pulse treatments, whereas succinic acid concentrations showed the opposite trend and were slightly higher under these conditions. In contrast, the pulse applied at 40 g/L CO₂ loss showed metabolite trends comparable to the oxygen saturation before inoculation (control), indicating no effect at this stage. Although large metabolic differences were observed between strains, these were consistent across all pulse treatments. Lactic acid was positively correlated with glycerol, succinic acid, and acetic acid across treatments.

### Central carbon metabolite yields under different oxygenation regimes

The main end-products of central carbon metabolism were evaluated by comparing metabolite yields (mol/mol) at the end of fermentation conducted under the different oxygenation regimes. These yields are shown in simplified metabolic pathways to illustrate how oxygen availability influenced carbon distribution (Fig. 4; Fig. S4 and S5).

#### Contrasting oxygenation treatments

Under the different oxygenation regimes, differences in metabolite yields were observed between strains; however, the overall response to oxygen availability was largely conserved. Biomass production under fully anaerobic conditions resulted in approximately threefold lower biomass yields than the oxygen saturation before inoculation (control), while continuous oxygenation produced the highest biomass yields, approximately three to four times higher than the oxygen saturation before inoculation (control) across all strains. Lactic acid yields showed the opposite trend, peaking under fully anaerobic conditions. Strain Y519 showed the strongest response: anaerobic fermentations produced approximately twice the lactic acid yield of the oxygen saturation before inoculation (control), while continuous oxygenation yielded no detectable lactic acid. For strain Y548, anaerobic and oxygen saturation before inoculation (control) conditions showed no significant difference, but continuous oxygenation reduced lactic acid yields fourfold compared to the control. Strain Y1240 produced no lactic acid under any condition. Ethanol yields followed a similar trend to lactic acid, with the highest yields obtained under fully anaerobic conditions (approximately 1.4 times higher than the oxygen saturation before inoculation [control]) and the lowest yields under continuous oxygenation (approximately 1.2 times lower than the oxygen saturation before inoculation [control]), with higher lactic acid-producing strains generally showing lower ethanol yields.

Glycerol yields showed no clear trend and remained relatively consistent across strains and oxygenation treatments. In contrast, succinic acid yields were highest under continuous oxygenation, ranging from approximately two to four times higher than the oxygen saturation before inoculation (control), and lowest under fully anaerobic conditions, with Y548 consistently producing the highest amounts followed by Y519, then Y1240. Acetic acid yields were generally highest under fully anaerobic conditions (approximately 1.5–3 times higher than the oxygen saturation before inoculation [control]) and lowest under continuous oxygenation (approximately 1.5–2 times lower than the oxygen saturation before inoculation [control]), with the exception of strain Y548, which produced substantially higher acetic acid under continuous oxygenation (approximately five times higher than the oxygen saturation before inoculation [control]).

Acetoin was only produced under continuous oxygenation conditions, where Y548 and Y1240 produced similar levels, while Y519 produced only trace amounts. Similarly, 2,3-butanediol was not produced under fully anaerobic conditions but was produced under continuous oxygenation at levels approximately 2.5–5 times higher than the oxygen saturation before inoculation (control) across all strains.

Metabolite yields were normalized to biomass (Fig. S4) to account for the substantial differences in biomass production observed between oxygenation treatments, allowing comparison of metabolic fluxes at an equivalent biomass. Overall, normalization resulted in clearer separation between treatments, indicating that the effect of oxygenation strategy on metabolite production per unit of biomass was more pronounced than suggested by molar yields alone. Following normalization to biomass (Fig. S4), the impact of oxygen treatment on lactic acid production was more pronounced, with significantly higher lactic acid production under fully anaerobic conditions for both producing strains and only trace amounts of lactic acid produced by Y548 under continuous oxygenation. Similarly, ethanol, glycerol, succinic acid, and acetic acid yields were all substantially higher under fully anaerobic conditions when expressed per unit of biomass. Notably, glycerol, which showed no clear trend in molar yields, exhibited increased production under anaerobic conditions after normalization, with similar trends observed between strains. In contrast to the molar yield data, succinic acid production was also highest under anaerobic conditions when expressed per unit of biomass, while maintaining similar strain trends to the molar yield data (Fig. 4). Acetoin production trends remained unchanged after normalization, whereas 2,3-butanediol now showed no significant differences between the oxygen saturation before inoculation (control) and continuous oxygenation treatments after normalization.

PCA was also performed on the end-product yields obtained under contrasting oxygenation conditions to provide an overall summary of treatment and strain effects (Fig. S6A). The PCA biplot explained 69.69% of the total variance, with PC1 accounting for 46.50% and PC2 accounting for 23.20% of the variance. Separation along PC1 was primarily driven by oxygenation treatment, while PC2 separated strains. All strains under fully anaerobic conditions clustered on the negative side of PC1 and were strongly associated with lactic acid and ethanol production. Strains under the oxygen saturation before inoculation (control) treatment clustered closer to the center of the plot and were not strongly associated with specific metabolites. Under continuous oxygenation, strains were positioned on the positive side of PC1 and were strongly associated with biomass, succinic acid, acetoin, and 2,3-butanediol production. Along PC2, strain differences were observed under continuous oxygenation, with Y548 positioned higher as it was more strongly associated with acetic acid and succinic acid, while Y519 and Y1240 clustered lower and were more strongly associated with biomass and 2,3-butanediol production. Glycerol and acetic acid were positioned near the center of the plot, indicating no strong association with a specific treatment or strain.

Carbon balance percentages for the contrasting oxygenation treatments are presented in Table S7. Carbon recovery for the oxygen saturation before inoculation (control) and fully anaerobic treatments was close to 100% for all strains, indicating that the measured metabolites and biomass accounted for most of the carbon consumed during fermentation. In contrast, carbon recovery under continuous oxygenation was substantially lower, ranging from approximately 66% to 72% across strains.

#### Timed oxygen pulse treatments

End-product yields (mol/mol) under timed oxygen pulse treatments are shown in Fig. S5. Overall, oxygen pulses had little effect on final metabolite yields. While clear differences were observed between the two phenotypically contrasting strains, the pattern of yields across pulse treatments was consistent within each strain, indicating that strain differences outweighed treatment effects. Lactic acid, ethanol, and glycerol yields showed no significant differences between the oxygen saturation before inoculation (control) and pulse treatments for both strains, indicating that pulses did not influence the final yields of these metabolites. The main treatment effects were observed for biomass, succinic acid, and acetic acid, where pulses applied at 20 h of fermentation and at 25% of maximum OD resulted in significant increases in biomass and succinic acid yields and significant decreases in acetic acid yields. In contrast, the oxygen pulse applied at 40 g/L CO₂ loss had no observable effect and produced end-product yields similar to the oxygen saturation before inoculation (control) treatment.

PCA supported these observations (Fig. S6B), explaining 82.64% of the total variance (PC1: 51.77%, PC2: 30.87%). Separation along PC1 was primarily driven by strain, with Y548 associated with higher overall metabolite production. In contrast, separation along PC2 reflected minor treatment effects, with pulses at 20 h of fermentation and 25% maximum OD associated with biomass and succinic acid, while the oxygen saturation before inoculation (control) treatment and the pulse applied at 40 g/L CO₂ loss clustered together and were associated with acetic acid.

Carbon recovery values for the pulse oxygenation treatments are presented in Table S7. Values were similar and close to 100% for both strains across all pulse treatments and the oxygen saturation before inoculation (control), indicating that timed oxygen pulses did not substantially affect overall carbon distribution, relative to the ratio of carbon directed toward biomass and metabolic product formation.

## DISCUSSION

### Oxygen-dependent metabolic strategy of *Lachancea thermotolerans*

This study demonstrates that oxygen availability fundamentally alters the metabolic strategy of *L. thermotolerans*, affecting fermentation performance, biomass formation, and carbon distribution. Across all strains, oxygen promoted a strong shift toward biomass production, consistent with previous studies (19, 20), accompanied by reduced ethanol and lactic acid yields and increased production of metabolites such as succinic acid, acetoin, and 2,3-butanediol. In contrast, strict anaerobic conditions resulted in limited growth and poor fermentation performance, indicating that *L. thermotolerans* retains a partial dependence on oxygen for efficient metabolism.

These observations confirm that *L. thermotolerans* is Crabtree-positive, as fermentation was maintained in the presence of oxygen (3, 27, 28). However, the substantial redirection of carbon toward biomass rather than ethanol is consistent with *L. thermotolerans* being a weakly Crabtree-positive species (3, 7). This behavior contrasts with *S. cerevisiae*, which maintains a predominantly fermentative metabolism even under aerobic conditions (29–31). The marked increase in biomass observed in this study (approximately 9- to 13-fold under aerobic conditions) highlights a fundamental difference in carbon allocation between these species (32).

This difference can be interpreted in an evolutionary context. Indeed, unlike *Saccharomyces*, *Lachancea* did not undergo whole-genome duplication, an event that is thought to have contributed to the strong fermentative capacity and efficient anaerobic growth of *Saccharomyces* species (29, 30). As a result, *L. thermotolerans* may retain a more ancestral metabolic configuration, characterized by a balance between respiratory and fermentative metabolism rather than a strong preference for fermentation. This is consistent with the poor fermentation performance observed under strictly anaerobic conditions in this study, in contrast to *S. cerevisiae*, which is well adapted to anaerobic environments and can sustain both biomass formation and completion of alcoholic fermentation in the absence of oxygen (32).

Phylogenetic analyses further support this interpretation, showing that *Lachancea* occupies an intermediate evolutionary position near the origin of anaerobic capability, alongside closely related genera such as *Torulaspora* (29). These yeasts have gained some capacity for anaerobic growth, but not to the same extent as *Saccharomyces*. Consistent with this evolutionary positioning, *Torulaspora* species have been reported to exhibit a similar oxygen-dependent reorientation of carbon flux toward biomass (33). The pronounced increase in biomass observed in *L. thermotolerans* under aerobic conditions in this study therefore reflects a metabolic strategy that is more closely aligned with *Torulaspora* than with *S. cerevisiae*.

A central finding of this study is that the increased biomass formation under aerobic conditions was not fully compensated by corresponding changes in fermentative metabolism. Ethanol and lactic acid yields decreased, and in parallel, moderate increases were observed for metabolites such as succinic acid, acetoin, and 2,3-butanediol, while glycerol production remained unchanged. Given the central role of glycerol in maintaining redox balance during fermentation, its lack of response to oxygen availability is notable. Indeed, in *S. cerevisiae*, glycerol production typically increases under aerobic conditions (32), whereas the absence of such a response in *L. thermotolerans* suggests a reduced reliance on this pathway for NAD^+^ regeneration. This is further supported by a previous study (34), which showed that glycerol production in *L. thermotolerans* was less affected by low nicotinic acid availability [i.e., the precursor of NAD(P)(H)] compared to *S. cerevisiae*, indicating a weaker dependence on glycerol formation for redox balance. Instead, alternative pathways, including increased flux through the TCA cycle and the production of acetoin and 2,3-butanediol, may contribute to redox balancing, although these appear insufficient to fully compensate for the metabolic shift. This is reflected in the carbon balance, which decreased substantially to approximately 70% under aerobic conditions, whereas values close to 100% were obtained for the other treatments. Together, these results indicate that the observed metabolic shifts were insufficient to account for the substantial increase in biomass and that a significant fraction of carbon remained unaccounted for. Most likely, part of the missing carbon resulted from increased respiratory activity under aerobic conditions, where a fraction of the sugars is fully oxidized, leading to additional CO₂ production that was not directly quantified in this study due to the open fermentation system required for oxygenation. This process would contribute to the energy (ATP) required for the increased biomass formation observed under these conditions (31). This incomplete compensation by metabolites suggests that carbon was redirected toward intracellular components that were not quantified. These likely include storage carbohydrates such as glycogen and trehalose, as well as lipids and structural components of the cell wall, all of which typically increase under aerobic growth conditions (35, 36). Enhanced flux toward these biosynthetic pathways would be consistent with the pronounced biomass formation observed and would reflect the higher energetic and anabolic demands associated with respiratory metabolism (31, 37). However, the magnitude of the imbalance indicates that this redistribution may not be sufficient to fully maintain metabolic homeostasis.

This interpretation is further supported by a rapid decline in cell density following peak OD under oxygenated conditions, while fermentation was still ongoing and excess fermentable carbon was still available, suggesting that not enough nutrients (other than the carbon sources) were available to the high biomass produced. This also indicates a possible redox imbalance between increased biomass production and cellular maintenance requirements under aerobic fermentation metabolism. An additional plausible explanation is the development of oxidative stress associated with increased respiratory activity and redox cycling. Although *L. thermotolerans* possesses a reactive oxygen species (ROS) detoxification toolkit similar to that of *S. cerevisiae*, its greater reliance on respiration under aerobic conditions may increase intracellular ROS formation and impose additional stress on cellular homeostasis (21). Importantly, cell viability was not directly assessed in this study, and future work should include viability and stress-response measurements to confirm whether the observed decline reflects cell death or reduced metabolic activity. Overall, these findings suggest that the strong oxygen-driven increase in biomass may not be sustainable over time, potentially due to limitations in redox and oxidative stress management.

Strain-dependent differences further highlight variability in metabolic strategies within *L. thermotolerans*. In particular, strain Y548 produced less biomass under oxygenated conditions than the other strains but appeared to compensate for reduced biomass formation by overproducing succinic acid and acetic acid, suggesting that the production of these compounds is linked to differences in acetyl-CoA management. This is consistent with a previous study (34), which demonstrated that acetyl-CoA management differs between *L. thermotolerans* and *S. cerevisiae*, as well as between *L. thermotolerans* strains. In contrast, the other strains did not show this response, potentially at the expense of metabolic stability. These differences indicate that carbon allocation and redox management strategies are strain-specific, reflecting underlying genetic diversity within the species.

### Lactic acid production: a facultative adaptive strategy

A defining feature of *L. thermotolerans* is the ability of certain strains to produce lactic acid, which introduces an additional dimension to its carbon metabolism (7, 10, 13). In this study, lactic acid production was demonstrated to be strongly dependent on oxygen availability, with high production under anaerobic conditions and near-complete repression under continuous aerobic conditions. This indicates that lactic acid production is not constitutive but rather a regulated response to environmental conditions.

Importantly, lactic acid production did not correlate directly with ethanol production, suggesting that it does not function simply as an alternative fermentative end-product. Instead, its role appears to be more complex. Under anaerobic conditions, where biomass formation is limited and respiration is inactive, lactic acid may serve as an additional pathway for NAD^+^ regeneration or as a means of redistributing carbon flux (3, 21, 38). This is supported by the fact that both lactic acid-producing strains responded similarly to oxygen availability but differed in magnitude, with Y519 producing substantially more lactic acid under anaerobic conditions, while Y548 showed a slight shift toward higher production of glycerol, succinic acid, and ethanol. However, the absence of increased glycerol production in the presence of oxygen, as previously observed in *S. cerevisiae* (32), suggests that redox balancing in *L. thermotolerans* is achieved through multiple pathways (e.g., succinic acid formation via the TCA cycle and the production of acetoin and 2,3-butanediol) rather than a single dominant mechanism.

The strong repression of lactic acid production in the presence of oxygen is associated with a pronounced shift in carbon flux toward biomass formation, and toward other metabolically linked pathways, as discussed above, thereby reducing carbon availability for lactic acid production. This raises the possibility that lactic acid production represents an overflow or auxiliary pathway that is activated under conditions where other metabolic routes are limited.

An alternative, but not mutually exclusive, explanation is that lactic acid production may function to limit ethanol accumulation under anaerobic conditions. Given that *L. thermotolerans* is considered less tolerant to high ethanol concentrations than *S. cerevisiae* (9, 23), the diversion of carbon toward lactic acid could represent a strategy to reduce ethanol production and mitigate its toxic effects. The repression of lactic acid production under aerobic conditions, where ethanol levels are already reduced, is consistent with this hypothesis.

Strain-dependent differences observed in the current study provide further insight into the role of lactic acid. The non-lactic acid-producing strain Y1240 consistently produced more biomass than lactic acid-producing strains, as well as less succinic acid and acetic acid regardless of the condition. This suggests that the absence of lactic acid production may reduce metabolic burden and allow more efficient carbon allocation toward growth, as supported by lower succinic acid production, suggesting reduced flux through the TCA cycle, and lower acetic acid production, which may indicate reduced acetyl-CoA overflow toward acetate formation (39–41). In contrast, lactic acid-producing strains diverted part of their carbon flux away from biomass and ethanol toward lactic acid and other metabolites associated with reduced growth efficiency, which together may impose a physiological cost.

From an ecological perspective, lactic acid production may confer a competitive advantage by acidifying the environment and inhibiting competing microorganisms (3, 7). In this context, it can be viewed as an adaptive trait that enhances ecological fitness under specific conditions. However, the reduced biomass and slower fermentation observed in lactic acid-producing strains in this study indicate that this advantage comes at a metabolic cost. This associated cost may limit its benefit under conditions where competition is less important or where efficient growth is prioritized, highlighting a trade-off between ecological competitiveness and metabolic efficiency that may explain why lactic acid production is retained in some strains but absent in others.

The timing of lactic acid production further supports its role as a regulated adaptive response. Its production was primarily associated with oxygen-limited conditions and was not directly linked to peak fermentation activity, suggesting that it is controlled independently of central fermentative pathways (21). This reinforces the idea that lactic acid production in *L. thermotolerans* is best described as a facultative adaptive strategy, which can be activated or repressed depending on environmental and physiological conditions. Previous studies have shown that this regulation is also influenced by factors such as pH, temperature, inoculation regime, and nitrogen availability; however, these generally exert a more indirect effect on lactic acid production, whereas sugar concentration (reflecting increased carbon availability) and oxygen availability (as supported by this study) represent more direct and stronger drivers of its production (3, 16–18, 21). In this study, lactic acid production functioned as a regulated metabolic feature that is activated under oxygen-limited conditions, where it may provide ecological or physiological benefits, but is repressed under conditions that favor efficient growth.

### Implications for oenological applications

From an applied perspective, this study evaluated whether oxygen availability can be used as a practical tool to regulate lactic acid production by *L. thermotolerans* during fermentation. Although some strains of this species are valued for their ability to contribute to wine bio-acidification, lactic acid production remains difficult to predict and control under winemaking conditions (7, 10). The use of oxygen as a regulatory factor therefore represents a potential strategy to improve consistency.

The use of contrasting oxygen conditions confirmed that oxygen availability is a major driver of lactic acid production but also highlighted clear practical limitations. Under fully anaerobic conditions, lactic acid yield increased substantially; however, this was accompanied by reduced biomass formation and impaired fermentation kinetics, making such conditions undesirable in an industrial context. In contrast, continuous oxygenation largely suppressed lactic acid production, with only trace amounts produced by strain Y548. While this confirms the strong regulatory effect of oxygen, continuous oxygen exposure is not a viable strategy in winemaking, as it not only eliminates the desired acidification effect but also increases the risk of oxidative spoilage and quality deterioration (42). This risk is particularly pronounced in later stages of fermentation, where reduced CO₂ production limits the displacement of oxygen, thereby enhancing the susceptibility of the wine to oxidation (42). Together, these findings demonstrate that while oxygen strongly influences lactic acid production, both extremes are impractical for oenological application.

To address this, oxygen pulse strategies were implemented to provide more realistic and controlled oxygen exposure. Overall, these treatments had limited impact on key fermentation parameters such as ethanol, glycerol, and notably lactic acid production. Although some treatment-specific effects were observed, including increased biomass and succinic acid production, as well as reduced acetic acid formation following earlier oxygen additions, these responses were not sufficient to achieve consistent or predictable modulation of lactic acid levels. In contrast, a late oxygen pulse had no measurable effect, suggesting that the timing of oxygen exposure is critical. These observations indicate that while oxygen availability clearly influences yeast metabolism, the pulse strategies applied in this study were insufficient to exert precise control over lactic acid production.

Nevertheless, the observed trends suggest that oxygen may still have potential as a tool to fine-tune metabolic behavior, provided that oxygen delivery strategies are further optimized. Given that lactic acid production is initiated early during the growth phase (3), earlier and potentially more sustained oxygen exposure may be required to effectively influence its production. Future work should therefore focus on refining the timing, intensity, and duration of oxygen addition to better align with the metabolic window during which lactic acid is produced.

Importantly, these experiments were conducted in synthetic grape juice, and the applicability of these findings to industrial winemaking conditions remains to be validated. The complexity of natural grape must, including its variable composition and microbial interactions, may influence the reproducibility of oxygen-driven effects. Further studies are therefore required to assess the impact of oxygen management strategies under realistic fermentation conditions and to determine their robustness at an industrial scale. In addition, a deeper understanding of the underlying molecular mechanisms governing the response of *L. thermotolerans* to oxygen will be essential to support the development of targeted and reliable control strategies.

Overall, this study demonstrates that while oxygen plays a central role in regulating lactic acid production, its practical application in oenology is not straightforward. Although oxygen pulses showed some potential to influence metabolic outcomes, their impact on lactic acid production was limited under the conditions tested. Consequently, achieving precise and predictable control of lactic acid production through oxygen management alone remains challenging but may be feasible with further optimization and validation under industrially relevant conditions.

## SUPPLEMENTAL MATERIAL

Fig. S1 aem.01155-26-s0001.tiff Fermentation performance of *Lachancea thermotolerans* strain Y548 under timed oxygen pulse treatments in synthetic grape juice (230 g/L sugar).

Fig. S2 aem.01155-26-s0002.tiff Heatmap representation of primary metabolite dynamics during alcoholic fermentation of *Lachancea thermotolerans* under contrasting oxygenation conditions in synthetic grape juice.

Fig. S3 aem.01155-26-s0003.tiff Heatmap representation of primary metabolite dynamics during alcoholic fermentation of *Lachancea thermotolerans* under timed oxygen pulse treatments in synthetic grape juice, including the two phenotypically contrasting strains, Y548 (high lactic acid producer) and Y1240 (non-lactic acid producer).

Fig. S4 aem.01155-26-s0004.tiff Simplified representation of central carbon metabolism in *Lachancea thermotolerans* during alcoholic fermentation under contrasting oxygenation conditions in synthetic grape juice.

Fig. S5 aem.01155-26-s0005.tiff Simplified representation of central carbon metabolism in *Lachancea thermotolerans* during alcoholic fermentation under timed oxygen pulse treatments in synthetic grape juice, including the two phenotypically contrasting strains, Y548 (high lactic acid producer) and Y1240 (non-lactic acid producer).

Fig. S6 aem.01155-26-s0006.tiff Principal component analysis (PCA) biplots of primary metabolites (mol/mol) measured at the end of alcoholic fermentation.

Supplemental legends aem.01155-26-s0007.docx Legends for Fig. S1 to S6.

Supplemental tables aem.01155-26-s0008.xlsx Tables S1 to S7.

Table S8 aem.01155-26-s0009.xlsx Raw fermentation kinetics (CO_2_ loss) data for *Lachancea thermotolerans* strains Y548, Y519, and Y1240 under contrasting oxygenation conditions and timed oxygen pulse treatments.

Table S9 aem.01155-26-s0010.xlsx Raw primary metabolite concentrations (g/L) measured throughout alcoholic fermentation for *Lachancea thermotolerans* strains Y548, Y519, and Y1240 under contrasting oxygenation conditions and timed oxygen pulse treatments.

ASM does not own the copyrights to Supplemental Material that may be linked to, or accessed through, an article. The authors have granted ASM a non-exclusive, world-wide license to publish the Supplemental Material files. Please contact the corresponding author directly for reuse.

## References

[1] Rogiers SY, Greer DH, Liu Y, Baby T, Xiao Z. 2022. Impact of climate change on grape berry ripening: an assessment of adaptation strategies for the Australian vineyard. Front Plant Sci 13:1094633. doi:10.3389/fpls.2022.1094633 PMC9811181 36618637

[2] Plantevin M, Merpault Y, Lecourt J, Destrac-Irvine A, Dijsktra L, van Leeuwen C. 2024. Characterization of varietal effects on the acidity and pH of grape berries for selection of varieties better adapted to climate change. Front Plant Sci 15:1439114. doi:10.3389/fpls.2024.1439114 PMC11499634 39450078

[3] Vicente J, Benito S, Marquina D, Santos A. 2025. Fermentative factors shape transcriptional response of *Lachancea thermotolerans* and wine acidification. NPJ Sci Food 9:97. doi:10.1038/s41538-025-00467-y PMC12144311 40481005

[4] Mira de Orduña R. 2010. Climate change associated effects on grape and wine quality and production. Food Research International 43:1844–1855. doi:10.1016/j.foodres.2010.05.001

[5] Payan C, Gancel AL, Jourdes M, Christmann M, Teissedre PL. 2023. Wine acidification methods: a review. OENO One 57:113–126. doi:10.20870/oeno-one.2023.57.3.7476

[6] van Leeuwen C, Sgubin G, Bois B, Ollat N, Swingedouw D, Zito S, Gambetta GA. 2024. Climate change impacts and adaptations of wine production. Nat Rev Earth Environ 5:258–275. doi:10.1038/s43017-024-00521-5

[7] Vicente J, Navascués E, Calderón F, Santos A, Marquina D, Benito S. 2021. An integrative view of the role of *Lachancea thermotolerans* in wine technology. Foods 10:2878. doi:10.3390/foods10112878 PMC8625220 34829158

[8] Benito S. 2018. The impacts of *Lachancea thermotolerans* yeast strains on winemaking. Appl Microbiol Biotechnol 102:6775–6790. doi:10.1007/s00253-018-9117-z 29876604

[9] Morata A, Loira I, Tesfaye W, Bañuelos MA, González C, Suárez Lepe JA. 2018. *Lachancea thermotolerans* applications in wine technology. Fermentation 4:53. doi:10.3390/fermentation4030053

[10] Vicente Javier, Vladic L, Navascués E, Brezina S, Santos A, Calderón F, Tesfaye W, Marquina D, Rauhut D, Benito S. 2024. A comparative study of *Lachancea thermotolerans* fermentative performance under standardized wine production conditions. Food Chem X 21:101214. doi:10.1016/j.fochx.2024.101214 PMC10876678 38379805

[11] Hranilovic A, Albertin W, Capone DL, Gallo A, Grbin PR, Danner L, Bastian SEP, Masneuf-Pomarede I, Coulon J, Bely M, Jiranek V. 2021. Impact of *Lachancea thermotolerans* on chemical composition and sensory profiles of merlot wines. Food Chem 349:129015. doi:10.1016/j.foodchem.2021.129015 33545601

[12] Hranilovic Ana, Albertin W, Capone DL, Gallo A, Grbin PR, Danner L, Bastian SEP, Masneuf-Pomarede I, Coulon J, Bely M, Jiranek V. 2022. Impact of *Lachancea thermotolerans* on chemical composition and sensory profiles of viognier wines. J Fungi 8:474. doi:10.3390/jof8050474 PMC9146010 35628730

[13] Jacobs TR, Rollero S, Delaherche A, Setati ME, Divol B. 2026. Uncovering genetic and phenotypic diversity connections in *Lachancea thermotolerans*: impact on wine aromatics. Food Microbiol 137:105048. doi:10.1016/j.fm.2026.105048 41722991

[14] Mucalo A, Budić-Leto I, Zdunić G. 2023. Effect of sequential fermentation with *Lachancea thermotolerans*/*S. cerevisiae* on aromatic and flavonoid profiles of plavac mali wine. Foods 12:1912. doi:10.3390/foods12091912 PMC10177817 37174449

[15] Fleming AJ, Threlfall RT. 2024. Using Non- *Saccharomyces* yeast to modify acidity during wine fermentations from *Vitis* hybrid grapes grown in a warm region. Am J Enol Vitic 75:0750004. doi:10.5344/ajev.2023.23012

[16] Gobbi M, Comitini F, Domizio P, Romani C, Lencioni L, Mannazzu I, Ciani M. 2013. *Lachancea thermotolerans* and *Saccharomyces cerevisiae* in simultaneous and sequential co-fermentation: a strategy to enhance acidity and improve the overall quality of wine. Food Microbiol 33:271–281. doi:10.1016/j.fm.2012.10.004 23200661

[17] Battjes J, Melkonian C, Mendoza SN, Haver A, Al-Nakeeb K, Koza A, Schrubbers L, Wagner M, Zeidan AA, Molenaar D, Teusink B. 2023. Ethanol-lactate transition of *Lachancea thermotolerans* is linked to nitrogen metabolism. Food Microbiol 110:104167. doi:10.1016/j.fm.2022.104167 36462823

[18] Joran A, Klein G, Roullier-Gall C, Alexandre H. 2022. Multiparametric approach to interactions between *Saccharomyces cerevisiae* and *Lachancea thermotolerans* during fermentation. Fermentation 8:286. doi:10.3390/fermentation8060286

[19] Shekhawat K, Bauer FF, Setati ME. 2017. Impact of oxygenation on the performance of three non-*Saccharomyces* yeasts in co-fermentation with *Saccharomyces cerevisiae*. Appl Microbiol Biotechnol 101:2479–2491. doi:10.1007/s00253-016-8001-y 27913851

[20] Shekhawat K, Porter TJ, Bauer FF, Setati ME. 2018. Employing oxygen pulses to modulate *Lachancea thermotolerans*–*Saccharomyces cerevisiae* chardonnay fermentations. Ann Microbiol 68:93–102. doi:10.1007/s13213-017-1319-6

[21] Shekhawat K, Bauer FF, Setati ME. 2020. The transcriptomic response of a wine strain of *Lachancea thermotolerans* to oxygen deprivation. FEMS Yeast Res 20:foaa054. doi:10.1093/femsyr/foaa054 32960268

[22] Kassier WE. 2023. Investigation and characterisation of lactic acid production by *Lachancea thermotolerans* MSc thesis, Stellenbosch University, Stellenbosch

[23] Porter TJ, Divol B, Setati ME. 2019. Investigating the biochemical and fermentation attributes of *Lachancea* species and strains: deciphering the potential contribution to wine chemical composition. Int J Food Microbiol 290:273–287. doi:10.1016/j.ijfoodmicro.2018.10.025 30412799

[24] Bely M, Sablayrolles JM, Barre P. 1990. Automatic detection of assimilable nitrogen deficiencies during alcoholic fermentation in oenological conditions. J Ferment Bioeng 70:246–252. doi:10.1016/0922-338X(90)90057-4

[25] Henschke PA. 1993. Metabolism of nitrogen compounds. In Fleet GH (ed), Wine microbiology and biotechnology. Harwood Academic Publishers, Chur, Switzerland.

[26] Hammer Ø, Harper DAT, Ryan PD. 2001. PAST: paleontological statistics software package for education and data analysis. Palaeontol Electron

[27] Dashko S, Zhou N, Compagno C, Piškur J. 2014. Why, when, and how did yeast evolve alcoholic fermentation? FEMS Yeast Res 14:826–832. doi:10.1111/1567-1364.12161 PMC4262006 24824836

[28] Schnierda T, Bauer FF, Divol B, van Rensburg E, Görgens JF. 2014. Optimization of carbon and nitrogen medium components for biomass production using non-*Saccharomyces* wine yeasts. Lett Appl Microbiol 58:478–485. doi:10.1111/lam.12217 24447289

[29] Hagman A, Säll T, Compagno C, Piskur J. 2013. Yeast “make-accumulate-consume” life strategy evolved as a multi-step process that predates the whole genome duplication. PLoS One 8:e68734. doi:10.1371/journal.pone.0068734 PMC3711898 23869229

[30] Hagman A, Piškur J. 2015. A study on the fundamental mechanism and the evolutionary driving forces behind aerobic fermentation in yeast. PLoS One 10:e0116942. doi:10.1371/journal.pone.0116942 PMC4305316 25617754

[31] Duncan JD, Setati ME, Divol B. 2025. The cellular symphony of redox cofactor management by yeasts in wine fermentation. Int J Food Microbiol 427:110966. doi:10.1016/j.ijfoodmicro.2024.110966 39536648

[32] Duncan JD, Devillers H, Camarasa C, Setati ME, Divol B. 2024. Oxygen alters redox cofactor dynamics and induces metabolic shifts in *Saccharomyces cerevisiae* during alcoholic fermentation. Food Microbiol 124:104624. doi:10.1016/j.fm.2024.104624 39244375

[33] Brandam C, Lai QP, Julien-Ortiz A, Taillandier P. 2013. Influence of oxygen on alcoholic fermentation by a wine strain of *Torulaspora delbrueckii*: kinetics and carbon mass balance. Biosci Biotechnol Biochem 77:1848–1853. doi:10.1271/bbb.130228 24018678

[34] Tyibilika V, Setati ME, Bloem A, Divol B, Camarasa C. 2024. Exploring fermentative metabolic response to varying exogenous supplies of redox cofactor precursors in selected wine yeast species. FEMS Yeast Res 24:foae029. doi:10.1093/femsyr/foae029 PMC11503943 39375837

[35] Klis FM, Boorsma A, De Groot PWJ. 2006. Cell wall construction in *Saccharomyces cerevisiae*. Yeast 23:185–202. doi:10.1002/yea.1349 16498706

[36] Shekhawat K, Patterton H, Bauer FF, Setati ME. 2019. RNA-seq based transcriptional analysis of *Saccharomyces cerevisiae* and *Lachancea thermotolerans* in mixed-culture fermentations under anaerobic conditions. BMC Genomics 20:145. doi:10.1186/s12864-019-5511-x PMC6379982 30777005

[37] Pfeiffer T, Morley A. 2014. An evolutionary perspective on the crabtree effect. Front Mol Biosci 1:17. doi:10.3389/fmolb.2014.00017 PMC4429655 25988158

[38] Sgouros G, Mallouchos A, Filippousi ME, Banilas G, Nisiotou A. 2020. Molecular characterization and enological potential of a high lactic acid-producing *Lachancea thermotolerans* vineyard strain. Foods 9:595. doi:10.3390/foods9050595 PMC7278797 32392718

[39] Cui Z, Zhong Y, Sun Z, Jiang Z, Deng J, Wang Q, Nielsen J, Hou J, Qi Q. 2023. Reconfiguration of the reductive TCA cycle enables high-level succinic acid production by *Yarrowia lipolytica*. Nat Commun 14:8480. doi:10.1038/s41467-023-44245-4 PMC10733433 38123538

[40] Korka V, Petropoulos A, Ioannidou SM, Lin CSK, Koutinas A, Fickers P. 2025. Harnessing yeasts for sustainable succinic acid production: advances in metabolic engineering and biorefinery integration. FEMS Yeast Res 25. doi:10.1093/femsyr/foaf052 PMC12501425 40972046

[41] Ding J, Holzwarth G, Penner MH, Patton-Vogt J, Bakalinsky AT. 2015. Overexpression of acetyl-CoA synthetase in *Saccharomyces cerevisiae* increases acetic acid tolerance. FEMS Microbiol Lett 362:1–7. doi:10.1093/femsle/fnu042 PMC4809976 25673654

[42] Tarko T, Duda-Chodak A, Sroka P, Siuta M. 2020. The impact of oxygen at various stages of vinification on the chemical composition and the antioxidant and sensory properties of white and red wines. Int J Food Sci 2020:7902974. doi:10.1155/2020/7902974 PMC7142352 32309422

