# Supplementary material for: Exploring Lachancea thermotolerans biodiversity: insights into lactic acid production and carbon metabolism in response to oxygen availability
Source: Appl Environ Microbiol. 2026 Aug 5;92(9):e01155-26. doi: 10.1128/aem.01155-26 (PMC13599520; doi:10.1128/aem.01155-26)
Supplement: Supplementary file 1 [file aem.01155-26-s0007.docx]

**Supplementary Figure captions:**

**FIG S1** Fermentation performance of Lachancea thermotolerans strain Y548 under timed oxygen pulse treatments in synthetic grape juice (230 g/L sugar). (**A**) Fermentation kinetics expressed as accumulated weight loss (g/L) due to CO₂ release, representing mean values of triplicate fermentations. (**B**) Yeast growth monitored by optical density at 600 nm (OD₆₀₀). Oxygen treatments included oxygen saturation before inoculation (control, blue), oxygen saturation before inoculation followed by an oxygen pulse at 20 h (yellow), or at 25% of maximum OD (purple), or at 40 g/L CO₂ loss (dark blue). Data points represent mean values of triplicate fermentations, and error bars indicate standard deviation. Standard deviation is not shown for fermentation kinetics due to the high-frequency automated weighing (every 20 min), which would overcrowd the figure. **Raw fermentation kinetics data underlying the CO₂ production curves are provided in Supplementary Table S8 (Fermentation kinetics raw data).**

**FIG S2** Heatmap representation of primary metabolite dynamics during alcoholic fermentation of *Lachancea thermotolerans* under contrasting oxygenation conditions in synthetic grape juice. All three genetically distinct strains are included and presented by oxygen treatment to allow comparison between strains within each treatment. Metabolite concentrations (g/L) were standardized using Z-scores to facilitate comparison between metabolites. Z-scores were calculated separately for each metabolite within each oxygenation condition using the mean and standard deviation across all strains and time points. Color intensity reflects relative metabolite abundance, with red indicating lower relative concentrations and blue indicating higher relative concentrations. Data are presented at selected fermentation stages corresponding to approximately 25% increments of total CO₂ loss, with the associated sampling time and actual CO₂ loss indicated. Pearson correlation analysis was performed using metabolite concentrations across all strains and time points within each oxygenation condition. Correlation coefficients are shown adjacent to the heatmaps, with bubble size proportional to the Pearson correlation coefficient and non-significant correlations (*p* > 0.05) are crossed out. **Raw metabolite concentration data underlying this figure are provided in Supplementary Table S9 (Primary metabolite raw data).**

**FIG S3** Heatmap representation of primary metabolite dynamics during alcoholic fermentation of Lachancea thermotolerans under timed oxygen pulse treatments in synthetic grape juice, including the two phenotypically contrasting strains, Y548 (high lactic acid producer) and Y1240 (non-lactic acid producer). The heatmaps allow comparison both between strains within a treatment (horizontal) and across treatments within a strain (vertical). Metabolite concentrations (g/L) were standardized using Z-scores to facilitate comparison between metabolites. Z-scores were calculated using the mean and standard deviation across all strains, oxygenation treatments, and time points, i.e. across the full dataset for each metabolite. Color intensity reflects relative metabolite abundance, with red indicating lower relative concentrations and blue indicating higher relative concentrations. Data are presented at selected fermentation stages corresponding to approximately 25% increments of total CO₂ loss, with the associated sampling time and actual CO₂ loss indicated. Pearson correlation analysis was performed using metabolite concentrations to assess relationships between strains within each oxygenation treatment, as well as between oxygenation treatments within each strain, following the comparative structure of the heatmaps.Correlation coefficients are shown adjacent to the heatmaps, with bubble size proportional to the Pearson correlation coefficient and non-significant correlations (*p* > 0.05) are crossed out. **Raw metabolite concentration data underlying this figure are provided in Supplementary Table S9 (Primary metabolite raw data).**

**FIG S4** Simplified representation of central carbon metabolism in *Lachancea thermotolerans* during alcoholic fermentation under contrasting oxygenation conditions in synthetic grape juice. Metabolite production is expressed as metabolite-to-biomass ratios at the end of alcoholic fermentation to account for large differences in dry biomass between oxygenation regimes and displayed as bar graphs integrated within the metabolic pathway. The biomass bar graph is shown transparently as biomass values were used to standardize metabolite production. Strains are grouped within each oxygen treatment. Bars represent mean values of triplicate fermentations, with error bars indicating standard deviation. Different letters above bars indicate statistically significant differences (*p* < 0.0001); uppercase letters denote differences within a strain across oxygen treatments, while lowercase letters denote differences between strains within the same oxygen treatment.

**FIG S5** Simplified representation of central carbon metabolism in *Lachancea thermotolerans* during alcoholic fermentation under timed oxygen pulse treatments in synthetic grape juice, including the two phenotypically contrasting strains, Y548 (high lactic acid producer) and Y1240 (non-lactic acid producer). Metabolite production and biomass is expressed as yield (mol product per mol sugar consumed) at the end of alcoholic fermentation and displayed as bar graphs integrated within the metabolic pathway. Strains are grouped within each oxygen treatment. Bars represent mean values of triplicate fermentations, with error bars indicating standard deviation. Different letters above bars indicate statistically significant differences (*p* < 0.0001); uppercase letters denote differences within a strain across oxygen treatments, and lowercase letters denote differences between strains within the same oxygen treatment.

**FIG S6** Principal component analysis (PCA) biplots of primary metabolites (mol/mol) measured at the end of alcoholic fermentation. Individual biological replicates are displayed. (**A**) PCA biplot of contrasting oxygenation treatments including all three genetically distinct strains, explaining 69.69% of the total variance, with principal components F1 (46.50%) and F2 (23.20%). Colors represent strains (Y548: green; Y519: blue; and Y1240: orange). (**B**) PCA biplot of timed oxygen pulse treatments including the two phenotypically contrasting strains (high and non-lactic acid producers), explaining 82.64% of the total variance, with principal components F1 (51.77%) and F2 (30.87%). Colors represent oxygen treatments (oxygen saturation before inoculation (control): light blue; O₂ pulse at 20 h of fermentation: yellow; O₂ pulse at 25% maximum OD: purple; and O₂ pulse at 40 g/L CO₂ loss: dark blue).
